# Infection and immunity: insights and therapeutic strategies through genomic analysis of the host, pathogen, and host–pathogen interaction

**DOI:** 10.1186/s13073-018-0583-9

**Published:** 2018-09-26

**Authors:** Julia Simundza

**Affiliations:** Genome Medicine, BMC, New York, NY 10004 USA

Several of the most celebrated milestones in medicine can be attributed to immunological research, stemming from Edward Jenner’s pioneering smallpox vaccinations and key contributions from Pasteur and Koch resolving the germ theory of infectious disease. Advances in the many years since have produced life-saving vaccines and anti-microbial therapeutics targeting a vast range of pathogens. Coupling these interventions with epidemiological insights into pathogen spread, evolution, and transmission has sharpened our ability to fight infection. Nevertheless, the modern era is still confronted with significant challenges. The emergence and re-emergence of viral pathogens such as Zika and Ebola, as well as an antimicrobial resistance crisis, pose serious global public-health threats and can lead to significant morbidity and mortality. In addition, key therapies such as successful human immunodeficiency virus (HIV)-1 or universal flu vaccines are still lacking, and a new focus on the microbiome has uncovered the consequential influence of broad-spectrum antibiotics, aimed at pathogenic microbes, on the commensal microbiome and human health [1]. These limitations call for refinement and innovation beyond current paradigms of understanding infection and immunity and developing effective therapies, via new methodologies, analytical vantage points, and collaborative research. This special issue of *Genome Medicine* highlights the ways in which state-of-the-art genomic-sequencing technologies, systems biology, and bioinformatics are advancing research into pathogens and the immune response, as well as applications in surveillance, detection, biomarker discovery, and therapeutic design to prevent and treat infection.

New insights into the immune system gained from high-resolution genomics approaches are facilitating the development of new immunotherapies for infectious disease, as well as autoimmunity and cancer. The diverse repertoire of adaptive immune cells, established by generating the sequence complexity of antigen-receptor genes through processes such as somatic hypermutation and VDJ recombination, is pivotal to the capacity of the immune system to respond to a multitude of antigens. Increasingly sensitive sequencing technologies and improved computational pipelines are making it easier to analyze sequence repertoires and their relationship to antigen specificity and clonotypic diversity. With applications toward vaccine design, this special issue features experimental and computational methods for optimizing paired-chain B cell immunoglobulin receptor sequencing. Warnatz and colleagues [2] present a technique to identify antigen-specific antibodies from single B cells by sequencing paired heavy- and light-chain mRNAs within isolated microsomes and using only common lab equipment. The BALDR pipeline from Bosinger and colleagues allows accurate de novo assembly and reconstruction of paired immunoglobulin sequences from single B cell transcriptomic data [3]. This might inform ‘systems vaccinology’ strategies to evaluate vaccine efficacy by identifying specific clonotypes over the course of a vaccine response and defining functional characteristics. As an example, BALDR has been used to characterize naïve cell B subclasses responsive to a germline-targeting eOD-GT8 immunogen, in a strategy to develop HIV-1 vaccines by priming naïve B cells with this immunogen such that they eventually produce VRC01-class broadly neutralizing antibodies [4].

In some cases, such as rapidly mutating viruses or active infections, it can be desirable or even necessary to look beyond vaccines for anti-viral strategies. Hoenen and colleagues [5] considered such an approach by screening for essential host factors involved in the Ebola virus life-cycle—targeting the host could overcome challenges in targeting the virus itself, and might also be applicable to other viruses using the same host pathways. Resulting from a novel genome-wide siRNA screen for Ebola host factors, the authors found that targeting the host pyrimidine synthetase pathway with an already FDA-approved compound produced effective anti-viral activity against Ebola as well as other viruses. Considering recent events in the Democratic Republic of Congo, Ebola outbreaks continue to be a global public-health concern, and although rapid surveillance and experimental vaccines are facilitating improved responses to outbreaks, development of such therapies could impact containment efforts [6].

Defining the mechanisms of resistance to current therapies is similarly as pressing as the development of new treatments. Winzeler and colleagues [7] analyze whole-genome sequencing data to distinguish isolates that lead to recurring or relapsing infections in patients infected with *Plasmodium vivax*, a malarial species prone to relapse because it can remain dormant within the host in a form that is refractory to typical medications. The study suggests that the applications of whole-genome sequencing in clinical infectious disease are many-fold—for example, as a potential diagnostic test that could influence treatment decisions, as an investigative tool to identify mechanisms of resistance by using pathogen genomic data, and as a source of population dynamics data to inform surveillance in regions of endemic infection.

Responses to Ebola virus and Zika virus outbreaks, among others, have demonstrated an essential role of genomics in strategies for surveillance and management of infectious diseases. Discussing avian influenza, Lam and Pybus [8] forecast that successful epidemic control “on a global scale will require increased genomic surveillance in poorly characterized regions, timely data sharing, and the development of new analytical methods to test hypotheses concerning influenza virus emergence and transmission.” Toward this, Borges et al. [9] have created INSaFLU, a bioinformatics platform for influenza virus sequencing data aimed toward increasing accessibility and analytical standardization across global research efforts. Such initiatives to maximize data and resource sharing to facilitate infectious disease research extend well beyond influenza—not only does the siRNA screening system presented by Hoenen and colleagues [5] provide the community with a minable dataset related to Ebola pathogenesis, but it also offers an experimental system that does not require biosafety level (BSL)-4, allowing similar experiments to be undertaken by many more labs.

Genomic and other high-throughput analyses are allowing an unprecedented level of analysis of human samples. Mark Davis and colleagues discuss emerging methods and translational insights gained from the human ‘model system’ for immunological research and consider how these are aiding our understanding of the complexities of human immunity [10]. One such approach is demonstrated by Khatri and colleagues [11], who, by analyzing human vaccine challenge datasets, found an association between symptom severity and expression of the *KLDR1* gene, leading them to propose that individual variation in *KLDR1*-expressing natural killer cell frequency could contribute to influenza susceptibility. A study of the *Milieu Intérieur* cohort [12] characterized variability in humoral responses to 15 different human pathogens in healthy individuals, and, along with age and sex, genome-wide analyses identified variation within human leukocyte antigen (HLA) and killer cell immunoglobulin-like receptor (KIR) loci that associated with particular antibody responses. Similar studies of natural variation in human immunity could bring about advances in ‘personalized immunology’, developing means to predict an individual’s risk of infection or other immune-related disease, response to therapy, and to guide tailored or precision therapies.

Each article in this issue uniquely leverages genomic data to innovate current approaches toward resolving mechanisms of pathogenesis, surveillance measures, and therapeutic design, and together the papers suggest that continued efforts are likely to impact research as well as clinical and public-health fronts. In addition to those opportunities described here, the integration of host and pathogen genomics has the potential to address central questions in the field such as the determination of human and zoonotic pathogen reservoirs and designing specific antimicrobial drugs to combat existing anti-microbial resistant strains, prevent future resistance, while sparing the commensal microbiome. As discussed by Alan Barrett, despite notable recent successes with known pathogens, the threat remains of an emergent unknown ‘disease X’, a response to which will require robust multidisciplinary platforms poised to fully characterize the pathogen and rapidly develop and validate an effective therapy [13]. To this effect, *Genome Medicine* continues to herald cutting-edge open-data and collaborative research initiatives that undoubtedly represent the future of this field.

## Abbreviations

BSL: Biosafety level
HIV: Human immunodeficiency virus
HLA: Human leukocyte antigen
KIR: Killer cell immunoglobulin-like receptor
